# Case report: A case report and literature review of extrapancreatic solid pseudopapillary neoplasm

**DOI:** 10.3389/fsurg.2022.1020044

**Published:** 2022-11-04

**Authors:** Hang Liu, Zhiquan Xu, Yaxu Wang, Haitao Gu, Yunhao Tang, Dabin Wu, Jijian Wang, Jianbo Zhang

**Affiliations:** Department of Gastrointestinal Surgery, The Second Affiliated Hospital of Chongqing Medical University, Chongqing, China

**Keywords:** diagnosis, extrapancreatic solid pseudopapillary neoplasm, ileum, origin, treatment

## Abstract

**Background:**

Solid pseudopapillary neoplasm (SPN) is a rare tumor with low malignant potential, which typically occurs in the pancreas. Extrapancreatic SPN is also extremely rare worldwide.

**Case presentation:**

We report a case of a 70-year-old woman hospitalized with abdominal pain and bloating. The patient did not have any underlying diseases, such as diabetes, coronary heart disease, or hypertension. More than 30 years ago, the patient underwent surgery for “ectopic pregnancy”. The patient had no family history of hereditary disease, nor did any immediate family members have a history of cancer. Laboratory tests showed that her hemoglobin and albumin levels were low and she had a high level of cancer antigen 125 (CA125). Enhanced computed tomography (CT) showed a large tumor in the abdomen and pelvis. The patient subsequently underwent surgery, and it was found that the tumor was attached to the terminal ileum. Pathological findings suggested that the tumor was an extrapancreatic SPN, with an ectopic pancreas found in the tumor tissue. The patient did not receive chemotherapy or radiotherapy after surgery. After 13 months of follow-up, the patient was admitted again with abdominal pain. CT showed tumor recurrence with extensive systemic metastases. The patient and her family refused reoperation and biopsy, and the patient was discharged after the abdominal pain and anemia resolved.

**Conclusion:**

We report a rare case of extrapancreatic SPN of ileal origin, which could be the first report worldwide. It had aggressive biological features, with recurrence and metastasis 13 months after surgery. For extrapancreatic SPN, the risk of recurrence should be assessed, and for tumors suspected of malignant behavior, a longer follow-up after discharge may be needed. Although SPN generally has a good prognosis after surgery, there is no consensus on whether postoperative chemotherapy and other treatments are needed for patients with high recurrence risk.

## Introduction

Solid pseudopapillary neoplasm (SPN) of the pancreas is an uncommon pancreatic tumor, accounting for approximately 0.3%–2.7% of all pancreatic tumors (1). In 2010, the WHO classified SPN as a low-grade malignant pancreatic tumor, although 10%–15% of SPN exhibit aggressive behavior, in rare cases resulting in patient death (2). The main clinical manifestations of SPN are abdominal pain, abdominal distension, and other discomforts caused by the enlargement of the tumor mass pressing on the abdomen. Although it has been reported in people aged 2–85, it is most common in women aged 20–40, with a female-to-male ratio of approximately 10:1 (3). Pancreatic SPN is a nomenclature to describe its histological features. It does not originate from pancreatic tissue.

Extrapancreatic primary SPN is extremely rare, and only approximately 50 cases have been reported (4–43). This article aimed to improve clinicians' understanding of extrapancreatic SPN, reduce the rate of missed diagnosis and delayed treatment, and ultimately maximize patient benefit. Here, we report a rare case of extrapancreatic SPN of ileal origin, which could be the first report worldwide. All previously published studies involving extrapancreatic SPN were reviewed. Written informed consent was obtained from the patient for the surgical intervention and case publication.

## Case presentation

**Figure 1:**
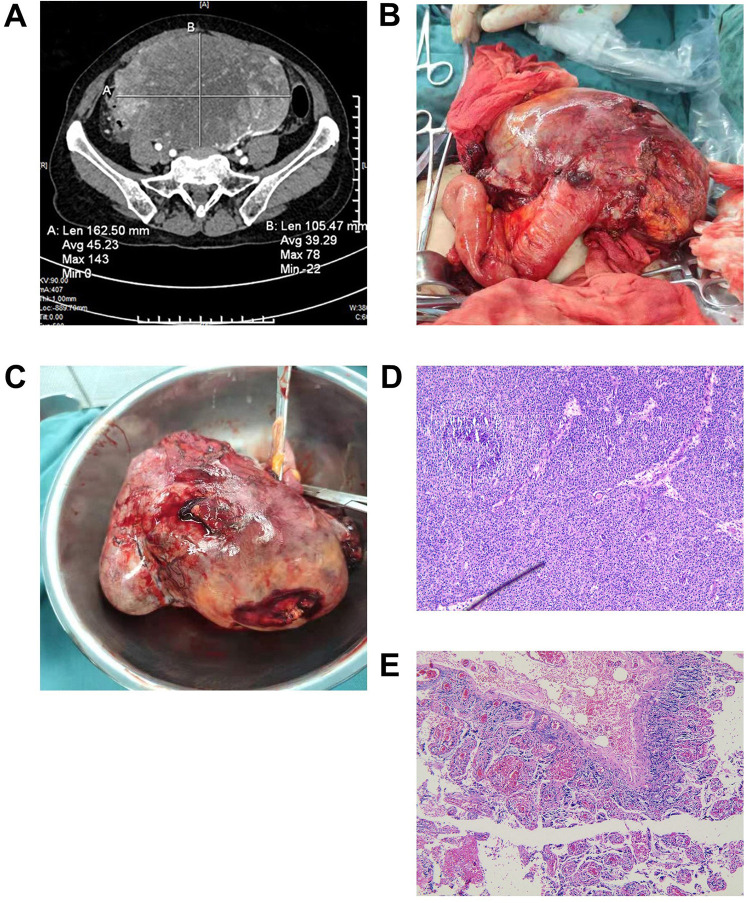
(**A**) An enhanced CT showed a huge mixed density mass in the lower abdominal cavity-pelvis. (**B**,**C**) Grossly, the tumor was attached to the terminal ileum, solid and cystic, with areas of necrosis and hemorrhage. (**D**) The tumor was histologically composed of cells arranged in the form of solid sheets and pseudopapillary areas (H & E, X 100). (**E**) The left adnexa showed the formation of white bodies in the left ovary (H & E, X 100). CT, computed tomography.

A 70-year-old woman was admitted to the hospital with sudden abdominal pain and bloating. The patient did not have any underlying diseases, such as diabetes, coronary heart disease, or hypertension. More than 30 years ago, the patient underwent surgery for “ectopic pregnancy”. The patient had no family history of hereditary disease, nor did any immediate family members have a history of cancer. Physical examination revealed mid-abdominal tenderness and a palpable 6 cm × 6 cm mass, with a firm texture and unclear boundary. Abdominal enhanced computed tomography (CT) showed a large mixed density mass in the lower abdominal cavity-pelvis, approximately 162.5 mm × 105 mm × 182 mm (Figure 1A). The mass was closely related to the right appendage, the adjacent bowel and bladder were compressed and moved, and no obvious obstruction or dilation of the bowel was seen. A cystic low-density, nonenhancing shadow was seen in the left adnexal area, measuring approximately 27 mm × 21 mm. No obvious abnormality was found in the liver, gallbladder, spleen, kidney, or pancreas. Laboratory tests showed that hemoglobin was 80 g/L (reference: 115–150 g/L), albumin was 25.9 g/L (reference: 40.0–55.0 g/L), and other routine laboratory tests showed no obvious abnormality. Furthermore, cancer antigen 19–9 (CA19–9) and carcinoembryonic antigen (CEA) were within normal limits, but CA125 was elevated to 706.7 U/ml (reference: 0.00–35.00 U/ml).

The patient had a definite diagnosis of a large tumor in the abdomen and pelvis. After communicating with the patient's family, we decided not to perform a preoperative biopsy and to surgically remove the tumor and perform a pathological biopsy. The patient underwent an exploratory laparotomy, and a large tumor in the abdomen and pelvis was revealed, approximately 20 cm × 20 cm in size. The tumor was attached to the terminal ileum, approximately 12 cm away from the ileocecal region, and could not be separated. We considered that the tumor probably originated in the small intestine (Figures 1B, C). The terminal mesentery was then segmented in a fan shape, and the intestinal tube was severed 3 cm distal to and 10 cm proximal to the tumor, followed by small bowel anastomosis. The pelvic cavity was explored again, and it was found that the right adnexa and uterus were atrophied. A cyst was observed in the left adnexa, approximately 3.0 cm × 3.0 cm in size, so the left adnexa was excised.

The postoperative recovery of the patient was uneventful, and she was discharged after half a month. The tumor was histologically composed of cells arranged in the form of solid sheets and pseudopapillary areas (Figure 1D). Ectopic pancreatic tissue was also observed histologically within the resected tumor. Immunohistochemistry (IHC) showed positive results for CD10, CD56, E-cadherin (weak positive), cytokeratin (CK), vimentin (Vim), progesterone receptor (PR), and succinate dehydrogenase complex subunit B (SDHB). In addition, the tumor cells showed nuclear staining for β-catenin, paranuclear dot-like staining for CD99, and focal staining for soluble protein-100 (S100) and synaptophysin (Syn). The proliferation index of Ki-67 was approximately 20%. The pathology of the left adnexa showed the formation of white bodies in the left ovary (Figure 1E). Combined with IHC, we confirmed that this was an extrapancreatic SPN of primary ileal origin.

**Figure 2:**
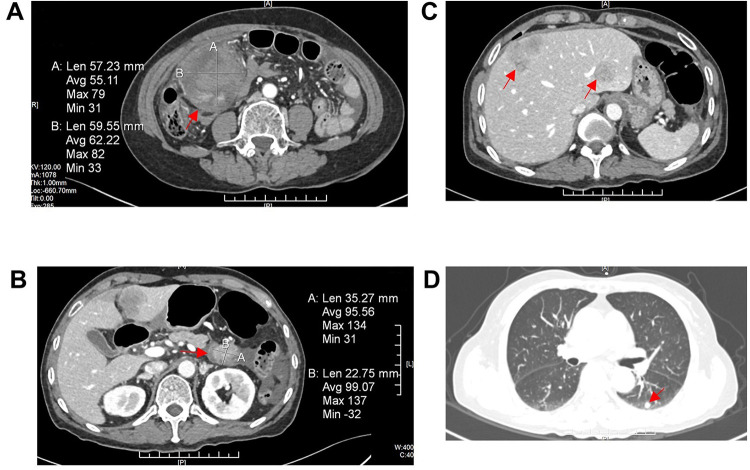
Enhanced CT of the chest and the whole abdomen showed tumor recurrence and extensive systemic metastases (**A**). There was a mixed density mass in the right middle abdomens, and the boundary was not clear (red arrowheads). (**B**) A cystic and solid mass was found next to the duodenum, irregular in shape, with unclear demarcation with the duodenum, and the solid part was significantly enhanced (red arrowheads). (**C**) There were several nodular and clump-shaped hypodense shadows in the liver, and the larger ones were located in the right lobe of the liver (red arrowheads). (**D**) There were multiple solid nodules in both lungs, and the largest one was located in the dorsal segment of the left lower lobe (red arrowheads). CT, computed tomography.

The patient did not receive chemotherapy or radiotherapy after surgery. Thirteen months after surgery, the patient was admitted again with abdominal pain. Laboratory examination showed her hemoglobin was 88 g/L, and her CA125 was elevated to 47.4 However, CA19–9 and CEA were within normal limits. Enhanced CT of the chest and the whole abdomen showed that there was a mixed density mass in the right middle abdomen, approximately 59 mm × 57 mm × 92 mm (left and right × front and back × up and down), and the boundary was not clear (Figure 2A). A cystic and solid mass was seen next to the duodenum, approximately 35.2 mm × 22.7 mm, with an irregular shape and unclear demarcation with the duodenum. The solid part was significantly enhanced (Figure 2B). There were several nodular and clump-shaped hypodense shadows in the liver, and the larger shadows were located in the right lobe of the liver (Figure 2C). There were multiple solid nodules in both lungs, and the largest nodule was located in the dorsal segment of the left lower lobe (Figure 2D). The patient and her family refused reoperation and biopsy, and the patient was discharged after her abdominal pain and anemia resolved.

## Discussion

We identified 50 cases with definite extrapancreatic SPN reported between 1990 and 2022 (including the case we reported). The characteristics of all cases are summarized in Table 1, based on the literature search (Supplementary Table S1 shows this in more detail). The most common sites of extrapancreatic SPN were the ovary (24.4%, 12/49), testis/paratestis (18.4%, 9/49), retroperitoneum (10.2%, 5/49), mesocolon (10.2%, 5/49), and omentum (10.2%, 5/49). Other rare sites included the stomach, liver, right adrenal, posterior mediastinum, mesentery, jejunum, and duodenum. Among the 50 patients, 16 were men, with a male-to-female ratio of 1:2.125. The average age of the patients was 39 years (range, 13–82 years), and tumors occurred mainly in people aged 20–40 (40%).

**Table 1:** Patient demographics and tumor characteristics of extrapancreatic solid pseudopapillary neoplasm.

Variable	Number (*n*)
Gender	
Male	16
Female	34
Age (year)	
Mean ± SD	39.1 ± 18.3
Symptoms	
Abdominal pain	16/37
Abdominal distension	9/37
Abdominal mass	6/37
Vomiting	4/37
Others	18/37
None	6/37
NA	13
Tumor location	
Ovary	12
Testis/paratestis	9
Retroperitoneum	5
Mesocolon	5
Omentum	5
Others	13
NA	1
Treatments	
Only surgery	41
Surgery + chemotherapy	5
Surgery + HIPEC	1
Surgery + herb medication	1
Surgery + molecularly targeted therapy	1
Antibiotic + antifungal therapy	1
Tumor size (cm)	
Mean ± SD	10.2 ± 7.5
Heterotopic pancreas	
Y	10
N	24
NA	16
Metastases	
Y	10
N	36
NA	4
Recurrence	
Y	8
N	30
NA	12
Follow-up (months)	
Mean ± SD	26.5 ± 32.8
Outcome	
NED	30
DOD	5
Alive with tumor	2
Died from severe sepsis	1
NA	12

Y, yes; N, no; NED, no evidence of disease; DOD, died of disease; NA, not available; HIPEC, hyperthermic intraperitoneal chemotherapy; SD, standard deviation.

The clinical symptoms of extrapancreatic SPN are often nonspecific, and some cases were even discovered incidentally during routine examinations. The symptoms are described in detail for 37 of the 50 patients. The clinical manifestations included abdominal pain in 16 cases (43.2%, 16/37), abdominal distension in 9 cases (24.3%, 9/37), abdominal mass in 6 cases (16.2%, 6/37), vomiting in 4 cases (10.8%, 4/37), and asymptomatic in 6 cases (16.2%, 6/37). A few patients had weight loss, nausea, fever, and fatigue. The patients' symptoms can also be different depending on the primary site, and they usually have more than one symptom. For example, menorrhagia, pelvic pain, and postmenopausal bleeding can occur in patients with SPN on the ovary. Laboratory tests of some patients may show decreased hemoglobin and increased white blood cells, but they are not specific. In addition, although serum markers (such as CEA, CA199, and CA125) can be increased, they are also not typical for diagnosing SPN (44). In our case, there was no change in CEA or CA-199. Although CA-125 was significantly elevated, the tumor originated in the ileum and was not an ovarian-related tumor.

In recent years, reports on SPN have gradually accumulated in various countries. However, there is still no clear conclusion about the origin of SPN. Some researchers found ectopic pancreatic tissue in the SPN tissue or at the tumor margin, so it is speculated that the SPN originated from this ectopic pancreatic tissue (16, 41–43). We found that in 50 cases of extrapancreatic SPN, only 10 (29.4%, 10/34) had ectopic pancreas, 24 (70.6%, 24/34) had no ectopic pancreas, and 16 did not mention it. Other researchers have found that during embryogenesis, the genital ridge is very close to the pancreatic primordium, so cells from the primordial gonad have the potential to migrate to the developing pancreas, thus leading to speculation that SPN may originate from germ ridge-related cells (45). This theory may also explain why extrapancreatic SPN tends to occur in the ovary and testis.

Imaging examinations play an important role in the initial diagnosis of SPN, among which the most commonly used imaging modalities are ultrasound, CT, and magnetic resonance imaging (MRI) (17, 46). The ultrasound features of SPN are mainly cystic and solid masses with heterogeneous internal echoes. Compared with ultrasound, CT can reveal the morphological structure of the entire tumor and the relationships between the surrounding tissues more clearly. It typically presents as a large mass of inhomogeneous density with solid and cystic components, with the solid component usually located at the margin of the mass and the cystic component in the center of the mass, often with an intact fibrous capsule. In addition, highly malignant SPN often exhibit local discontinuities of the capsule, unclear edges, and relatively large tumor volumes. On MRI, the solid component and cystic component have different signal responses. T1-weighted imaging mostly shows heterogeneously mixed signals, T2-weighted imaging is iso- or slightly hyperintense, and diffusion-weighted imaging is hyperintense. In addition, the presence of solid and cystic components on MRI with hemorrhage but no septum should be highly suspicious for SPN. Among the 50 cases of extrapancreatic SPN, CT was the most widely used diagnostic method. In our case, the tumor's location, invasion, and metastasis were also clarified by contrast-enhanced CT.

Pathological examination and IHC are the most reliable methods for diagnosing SPN. The average size of surgical resection specimens among the 50 cases was approximately 10.2 ± 7.5 cm (0.5–30 cm). The growth pattern of SPN is more diverse, and it can manifest as solid, pseudopapillary, and cystic structures in different proportions. Microscopically, one or more layers of tumor cells are arranged around the fibrovascular axis to form pseudopapillary protrusions, which are typical pathological features (9). IHC showed that almost all tumors were positive for β-catenin (nuclear staining), CD10, CD56, and vimentin. Most tumors were positive for CD99, α1-antitrypsin, NSE, P504s, and PR; some were positive for synaptophysin. However, they usually do not express chromogranin A or E-cadherin (3, 46). Based on these typical features, the diagnosis of SPN can be performed.

In the literature we reviewed, a total of 49 patients with extrapancreatic SPN received surgery, and one patient received antibiotic and antifungal therapy due to severe infection. Among 50 cases of extrapancreatic SPN, five patients received chemotherapy, one received molecularly targeted therapy (the drug was imatinib), one received herbal medication, and one received HIPEC. However, the current number of cases is too small, and more clinical evidence is needed to evaluate the effect of adjuvant therapy on SPN. Ten patients with extrapancreatic SPN had tumor metastasis (21.7%, 10/46), 36 had no tumor metastasis (78.3%, 36/46), and 4 had no mention. Among the 50 patients with extrapancreatic SPN, 38 patients had follow-up information, and the follow-up time ranged from 3 to 144 months. There were 8 patients (21.1%, 8/38) with tumor recurrence, 30 (78.9%, 30/38) without recurrence, and 12 who did not mention recurrence. There were 38 patients with clear outcomes, and 32 patients (84.2%, 32/38) survived well, including 30 patients with no evidence of disease (NED) and 2 patients alive with tumors. There were six deaths (15.8%, 6/38), of which five died of SPN recurrence, and one died of severe sepsis.

Pancreatic SPN is a low-grade malignant tumor; only 15% will develop metastasis, and a long-term survival rate is observed in more than 95% of the cases. The metastasis rate of extrapancreatic SPN was 21.7%, and the survival rate of 38 patients with follow-up was 84.2%. Therefore, extrapancreatic SPN may have a favorable clinical course similar to that of SPN. Although the liver is the most common site of distant metastases, the mesentery, mentum, peritoneum, and lungs may also be involved. However, metastasis or invasion of adjacent organs is not a contraindication to surgery, and the patients could also have a longer survival time after reoperation (46). In addition to surgery, conventional adjuvant treatments can also be used for the treatment of SPN, such as hyperthermic intraperitoneal chemotherapy (HIPEC), radiofrequency ablation (RFA), transcatheter arterial embolization, radiotherapy, and chemotherapy (19).

Extrapancreatic SPN is a rare low-grade malignancy with a good overall prognosis. It mainly occurs in the ovary, testis/paratestis, retroperitoneum, mesocolon, and omentum. Abdominal pain, bloating, and a palpable mass are the most common symptoms. Laboratory tests and serum tumor markers are usually nonspecific. Immunohistochemical staining of biopsy or the surgical resection specimen is the main method for diagnosis and differential diagnosis. The most unique immunohistochemical marker is the abnormal nuclear staining of β-catenin. The best treatment for extrapancreatic SPN is still radical surgical resection. Even in the case of tumor metastasis, most patients can still be radically cured by surgical resection of the primary tumor and metastases because of the slow clinical progression after metastasis. For unresectable patients, there is limited evidence to support other treatments, such as chemotherapy.

Primary SPN occurring outside the pancreas are exceedingly rare. Our case is unique because this is the first reported extrapancreatic SPN of ileal origin. Due to its abnormal location, an accurate diagnosis was a challenge for pathologists. Another feature that makes this case unique is that the patient developed extensive lung and abdominal metastases 13 months after surgery. Some doctors have proposed risk criteria for recurrence after SPN, including diffuse tumor growth, capsular involvement, vascular or perineural invasion, lymph node metastasis or distant metastasis, and a Ki-67 index ≥4% is associated with SPN recurrence. Intraoperative tumor rupture may be the cause of peritoneal recurrence (21). Some researchers also found that the tumor size for cases with metastases was larger than that of nonmetastatic tumors (8.13 ± 1.03 cm for metastatic tumors and 5.20 ± 3.78 cm for nonmetastatic, range 7–9 cm, *P* < 0.012). Larger tumor size was significantly associated with the risk of metastasis and recurrence (*P *< 0.002) (46). The tumor resected from our patient was approximately 20 cm × 20 cm in size, and the postoperative pathological finding of the Ki-67 index was approximately 20%, so we speculated that these clinical features of the patient were one of the possible reasons for the recurrence. In addition, our patient only underwent surgery and did not receive other treatments, such as chemotherapy or radiotherapy. Although SPN generally has a good prognosis after surgery, there is no consensus on whether postoperative chemotherapy and other treatments are needed for patients with high recurrence risk. Whether this is related to postoperative recurrence also needs further research.

## Conclusion

We report a rare case of extrapancreatic SPN of ileal origin, which could be the first report worldwide. It had aggressive biological features, with recurrence and metastasis 13 months after surgery. For extrapancreatic SPN, the risk of recurrence should be assessed, and for tumors suspected of malignant behavior, a longer follow-up after discharge may be necessary. Our case may extend our understanding of the biological behavior of extrapancreatic SPN and provide clinical experience with the diagnosis and treatment of extrapancreatic SPN to avoid a missed diagnosis and delayed treatment.

## Data Availability

The original contributions presented in the study are included in the article/**Supplementary Material**, further inquiries can be directed to the corresponding author/s.
