# Supplementary material for: Case report: A case report and literature review of extrapancreatic solid pseudopapillary neoplasm
Source: Front Surg. 2022 Nov 4;9:1020044. doi: 10.3389/fsurg.2022.1020044 (PMC9672344; doi:10.3389/fsurg.2022.1020044)
Supplement: Supplementary file 1 [file Table1.docx]

**Supplementary Table S1.** Patient demographics and tumor characteristics of extrapancreatic SPN.

| **Age** | **Sex** | **Location** | **Size(cm)** | **Symptom** | **Treatment** | **Heterotopic pancreas** | **Metastases** | **Recurrence** | **Follow-up** | **Outcome** | **Ref.** |
| --- | --- | --- | --- | --- | --- | --- | --- | --- | --- | --- | --- |
| 37 | F | Right adrenal | 8.1 | None | Surgery | N | N | NA | NA | NA | 4 |
| 14 | F | Gastric antrum | 5 | Abdominal pain & vomiting | Chemotherapy & Surgery | N | N | N | 2 years | NED | 5 |
| 25 | M | Beneath the transverse mesocolon | 8 | Abdominal mass | Surgery | Y | N | N | 2 years | NED | 6 |
| 16 | F | Jejunum | 7.5 | Abdominal pain | Surgery | Y | N | N | 6 months | NED | 7 |
| 45 | M | Greater omentum | 18 | Abdominal mass | Molecularly targeted therapy & Surgery | N | Y | Y | 8 years | DOD | 8 |
| 78 | M | Greater omentum | 16 | Abdominal pain & abdominal mass & | Surgery | N | Y | Y | 10 months | NA | 9 |
| 45 | F | Right ovary | 7.5 | weight loss abdominal pain & distension | Chemotherapy & Surgery | N | Y | N | 8 months | DOD | 10 |
| 30 | F | Retroperitoneum | 15 | Abdominal pain & abdominal mass | Surgery | N | N | N | 1 years | NED | 11 |
| 41 | F | Liver | 30 (right) 5.5 (left) | Abdominal distension & pain | Herb medication & Surgery | N | N | N | 13 months | NED | 12 |
| 15 | F | Mesocolon | 21 | Abdominal distension & pain | Surgery | Y | N | NA | NA | NA | 13 |
| 18 | M | Mesocolon | 6 | Abdominal Pain & altered bowel habits | Surgery | Y | N | NA | NA | NA | 14 |
| 18 | F | Mesocolon | 5 | Abdominal pain& nausea& vomiting | Surgery | N | N | NA | NA | NA | 15 |
| 33 | M | Root of small bowel mesentery | 6 | Abdominal distension & pain & Vomiting | Chemotherapy & Surgery | Y | Y | N | 10 months | NED | 16 |
| 36 | F | Retroperitoneum | NA | None | Surgery | NA | N | N | 8 months | NED | 17 |
| 25 | F | Right ovarian | 16.5 | Menorrhagia & abdominal fullness | Surgery | NA | N | N | 12 years | NED | 18 |
| 61 | F | NA | 22.7 | Abdominal pain & fever | Antibiotic & Antifungal therapy | NA | Y | NA | NA | Died from severe sepsis | 19 |
| 22 | F | Retroperitoneum | 6 | None | Surgery | Y | N | N | 14 months | NED | 20 |
| 40 | M | Mesentery | 25 | Abdominal distention | HIPEC & Surgery | NA | N | Y | 4 years | DOD | 21 |
| 40 | F | Left ovary | 20 | Weight loss | Chemotherapy & Surgery | N | Y | Y | 20 months | DOD | 22 |
| 50 | F | Mesentery | 19 | Abdominal pain | Surgery | NA | N | N | 2 years | NED | 23 |
| 49 | F | Right ovary | 4.5 | Pelvic pain & postmenopausal bleeding | Surgery | N | N | N | 1 years | NED | 24 |
| 56 | F | Liver | 20 | Cholestasis & hepatic mass | Surgery | N | N | N | 1 years | NED | 25 |
| 18 | F | Right ovary | 9.5 | Abdominal distension | Surgery | N | N | NA | NA | NA | 26 |
| 32 | M | paratestis | 4.8 | NA | Surgery | NA | N | N | 3 months | NED | 27 |
| 18 | F | left ovarian | 17.5 | NA | Surgery | N | N | N | 3 months | NED | 28 |
| 48 | F | left ovarian | 8 | Left lower-quadrant discomfort & left back pain | Surgery | N | N | N | 9 months | NED | 29 |
| 32 | F | pyloric region of the stomach | 10 | NA | Chemotherapy & Surgery | N | Y | Y | 10 years | Alive with tumor | 30 |
| 73 | M | duodenum& | 10 | Abdominal mass & vomiting & fatigue & weight loss | Surgery | N | Y | Y | 3 months | DOD | 30 |
| 47 | F | Retroperitoneum | 16 | Abdominal pain | Surgery | N | NA | N | 14 months | NEDn | 31 |
| 39 | F | right ovary | 6 | Abdominal pain | Surgery | NA | N | Y | 3 years | NED | 32 |
| 46 | F | greater omentum | 5.2 | None | Surgery | N | N | N | 3 months | NED | 33 |
| 17 | F | left ovary | 25.5 | Abdominal mass | Surgery | N | N | N | 6 years | NED | 34 |
| 57 | F | right ovary | 3 | None | Surgery | N | N | NA | NA | NA | 34 |
| 21 | F | left ovary | 14 | Abdominal distension | Surgery | N | N | NA | NA | NA | 34 |
| 22 | F | retroperitoneum | 7 | Abdominal discomfort | Surgery | N | N | N | 6 months | NED | 35 |
| 62 | F | posterior mediastinum | 3 | None | Surgery | N | N | N | 5 months | NED | 36 |
| 67 | M | testis | 0.5 | NA | Surgery | NA | N | N | 6 years | NED | 37 |
| 38 | M | testis | 2.1 | NA | Surgery | NA | N | N | 3 years | NED | 37 |
| 33 | M | paratestis | 0.5 | NA | Surgery | NA | N | N | 3years | NED | 37 |
| 24 | M | testis | 3 | NA | Surgery | NA | N | N | 2years | NED | 37 |
| 82 | M | testis | 2 | NA | Surgery | NA | N | NA | NA | NA | 37 |
| 37 | M | testis | 1 | NA | Surgery | NA | N | N | 3 months | NED | 37 |
| 60 | M | testis | 1.5 | Hydrocele | Surgery | NA | N | N | 15 months | NED | 38 |
| 56 | M | testis | 3.2 | Hydrocele | Surgery | NA | N | N | 27 months | NED | 38 |
| 53 | F | lesser Omentum | 7.5 | NA | Surgery | N | N | N | 7 months | NED | 39 |
| 25 | F | right ovary | 12 | Abdominal pain | Surgery | NA | Y | N | 18 months | NED | 40 |
| 13 | F | mesocolon | 8 | NA | Surgery | Y | NA | NA | NA | NA | 41 |
| 33 | F | mesocolon | 6 | NA | Surgery | Y | NA | NA | NA | NA | 42 |
| NA | F | Omentum | NA | NA | Surgery | Y | NA | NA | NA | NA | 43 |
| 70 | F | Ileum | 20 | Abdominal distension & pain | Surgery | Y | Y | Y | 13 months | Alive with tumor | present case |

*Y: Yes, N: No, NED: No evidence of disease; DOD: Died of disease; NA: Not available; HIPEC: Hyperthermic intraperitoneal chemotherapy; Ref: reference*
